# Neuroprotective Effect of Methylene Blue in a Rat Model of Traumatic Optic Neuropathy

**DOI:** 10.3390/ph18060920

**Published:** 2025-06-19

**Authors:** Nicolás S. Ciranna, Ronan Nakamura, Rafael Peláez, Álvaro Pérez-Sala, Patricia Sarrión, Juan C. Fernández, Alejandra Paganelli, Agustín P. Aranalde, Ulises P. Ruiz, Juan J. López-Costa, César F. Loidl, Alfredo Martínez, Manuel Rey-Funes

**Affiliations:** 1 Institute of Cell Biology and Neurosciences “Prof. E. de Robertis”, Faculty of Medicine, University of Buenos Aires, Buenos Aires 1821, Argentina; nsc26@hotmail.com (N.S.C.); ronannak7@gmail.com (R.N.); docfer2015@gmail.com (J.C.F.); apaganelli@fmed.uba.ar (A.P.); aranalde261@gmail.com (A.P.A.); ulisesruiz604@gmail.com (U.P.R.); jjlopez@fmed.uba.ar (J.J.L.-C.); cfloidl@yahoo.com.ar (C.F.L.); mreyfunes@fmed.uba.ar (M.R.-F.); 2 Biomarkers and Molecular Signaling Group, Neurodegenerative Diseases Area, Center for Biomedical Research of La Rioja (CIBIR), 26006 Logroño, Spain; aperez@riojasalud.es (Á.P.-S.); psarrion@riojasalud.es (P.S.); 3 Angiogenesis Group, Oncology Area, Center for Biomedical Research of La Rioja (CIBIR), 26006 Logroño, Spain

**Keywords:** optic neuropathy, trauma, neuroprotection, methylene blue, electroretinography, retinal ganglion cells, neurodegeneration

## Abstract

**Background**: Traumatic optic neuropathy (TON) represents a major cause of vision loss worldwide, and treatment options are limited. Here, we study whether methylene blue (MB), a free radical scavenger, is able to prevent morphological and electrophysiological hallmarks of neuropathy in an animal model of TON. **Methods**: The left eyes of Wistar rats were subjected to intraorbital nerve crush (IONC) while the right ones were sham operated. The group of rats treated with MB (n = 16) received five intraperitoneal injections with 2.0 mg/kg MB in the 24 h following IONC while the control group (n = 16) received just vehicle (PBS) as a control. Twenty-one days after surgery, scotopic full field (scERG), scotopic oscillatory potentials (OP), photopic full field (phERG) and pattern (PERG) electroretinography were performed for retinal function assessment. Furthermore, the number of cell nuclei in the ganglion cell layer (GCL) was recorded in post mortem histological sections. **Results**: IONC induced very significant reductions in electrophysiological parameters including scotopic a- and b-wave, OPs, photopic b-wave, PhNR amplitude and N2 amplitude. In addition, it also generated a significant prolongation of the N2 implicit time, indicating a profound impact on retinal function. This was further corroborated by a very significant reduction in the number of neuronal nuclei in the GCL, suggesting an intense loss and functional impairment of retinal ganglion cells. MB treatment was able to prevent, partially or completely, all those parameters, indicating the efficiency of such approach. **Conclusions**: Since MB is already approved for clinical use and presents a high safety profile, it could be repurposed as a neuroprotective drug for ophthalmological applications once proper phase 2 clinical trials are accomplished.

## 1. Introduction

Traumatic injuries are one of the most prevalent causes of disability and death worldwide, especially in young individuals. Many health complications can arise from traumatism, one of them being vision loss, which is very common because of indirect blunt trauma causing traumatic optic neuropathy (TON) [1]. In the context of a trauma patient, TON should always be suspected, given that lesions to the optic nerve could potentially translate into irreversible blindness, so immediate treatment is crucial to prevent this scenario [2].

The reported incidence of TON ranges from 0.7 to 2.5% in patients suffering from head or facial trauma [3], while it ranges from 0.5 to 5.0% in individuals with traumatic brain injury (TBI) [4]. Prevalence of TON has not been established worldwide, but a recent UK study has reported a minimum prevalence of 1 in 1,000,000 in the general population, being similar for both adults [5] and children [6]. The most common causes of TON are motor vehicle accidents, falls, assaults and sport injuries [7], yet other causes can be responsible in different situations, such as child abuse [8] or blast injuries in the battle ground [9,10,11]. Furthermore, TON can also have iatrogenic causes through complications of maxillofacial surgery or endoscopic sinus procedures [12].

Trauma to the optic nerve can eventually lead to retinal ganglion cell (RGC) death which ultimately causes several degrees of blindness, none of them reversible with current treatment options [1,13]. Many experimental protocols are being investigated to achieve neuroprotection to the retina and optic nerve. These include up-regulation of mTOR, mesenchymal stem cell transplantation, gene therapy (e.g., OPTN overexpression or NFATc4 silencing), stimulation of the Nfe2l3 and BDNF/TrkB pathways, administration of carotenoid derivatives, and combined treatments with ripasudil and brimonidine [14,15,16,17,18,19,20,21,22]. However, there is still a long way to go until an approved and effective treatment is available for these patients.

Over the last decade, our research team has studied many neuroprotective strategies to preserve vision in different models of retinopathy and neuropathy. Among them, the intraorbital nerve crush (IONC) model was utilized to test the efficacy of therapeutic hypothermia [23] and hypothermia mimetic molecules [24]. In recent years, methylene blue (MB), a guanylyl cyclase inhibitor and free radical scavenger [25], which inhibits both the constitutive and inducible isoforms of NOS [26,27], has been proven to be neuroprotective in experimental models of perinatal asphyxia-induced retinopathy [28,29] and glaucoma [30]. Since nitric oxide (NO) is a known mediator of TON [31,32,33], we hypothesize that MB could be a promising new treatment, applied shortly after trauma, for those patients whose vision health may be compromised or at risk.

The main objective of this work, therefore, was to demonstrate the neuroprotective properties of MB to prevent RGC loss and detriment of retinal electrophysiology following trauma to the optic nerve. For that purpose, the pharmacological effects of MB were evaluated in a rat model of IONC through the evaluation of several electroretinography paradigms and the follow up of morphological and molecular changes in the retina.

## 2. Results

### 2.1. Surgery and Treatment Groups

**Figure 1:**
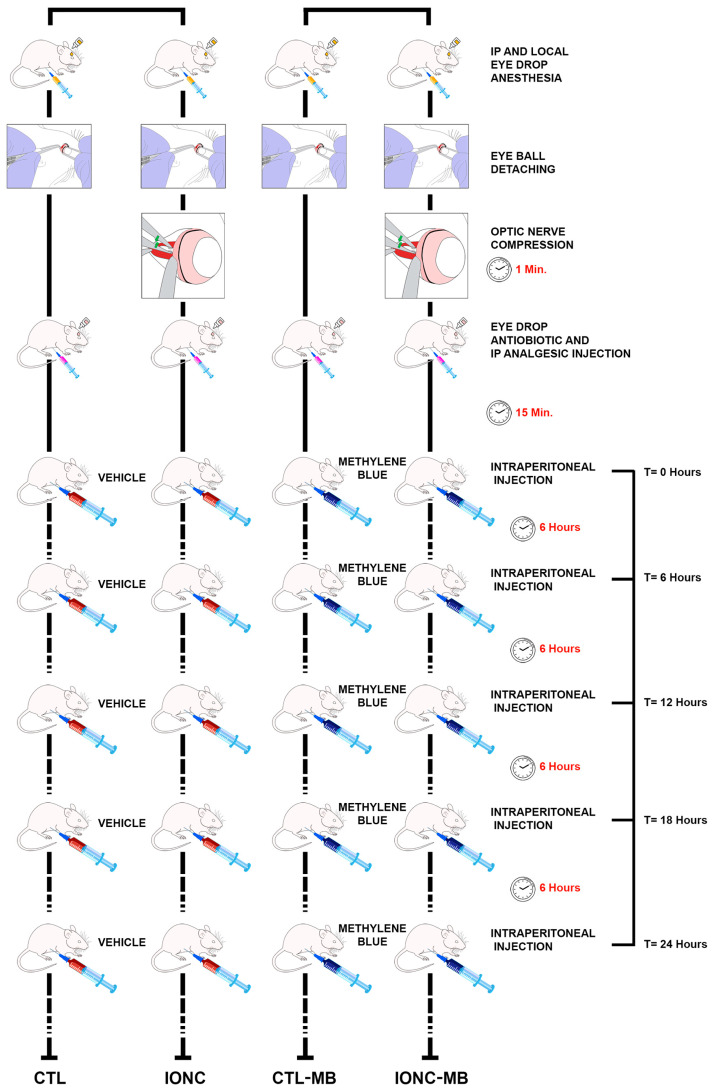
Schematic drawing of the experimental procedure. The left eyes of all animals (n = 32 rats) were subjected to intraorbital optic nerve crush (IONC) while the right eyes were sham-operated. Then, rats were injected intraperitoneally with either PBS or 2.0 mg/kg MB at 0, 6, 12, 18 and 24 h post-surgery. As a result, four experimental groups of eyes were generated: CTL, IONC, CTL-MB and IONC-MB (n = 16 eyes per group). Twenty-one days after surgery, all animals were subjected to diverse ERG tests and sacrificed.

Immediately after surgery, 16 animals started intraperitoneal treatment with 2.0 mg/kg MB, while the other 16 rats received the same volume of vehicle (PBS). This design generated four experimental eye groups: (i) sham-operated, injected with vehicle (CTL), (ii) IONC-operated, injected with vehicle (IONC), (iii) sham-operated, injected with MB (CTL-MB) and (iv) IONC-operated, injected with MB (IONC-MB) (Figure 1).

### 2.2. Methylene Blue Restores Electroretinogram Patterns

Twenty-one days after surgery, all animals were subjected to electrophysiological evaluation with one of three different paradigms: scotopic electroretinography (scERG, n = 8 per group), photopic electroretinography (phERG, n = 8 per group) or pattern electroretinography (PERG, n = 8 per group).

#### 2.2.1. Scotopic Full Field Electroretinography

**Figure 2:**
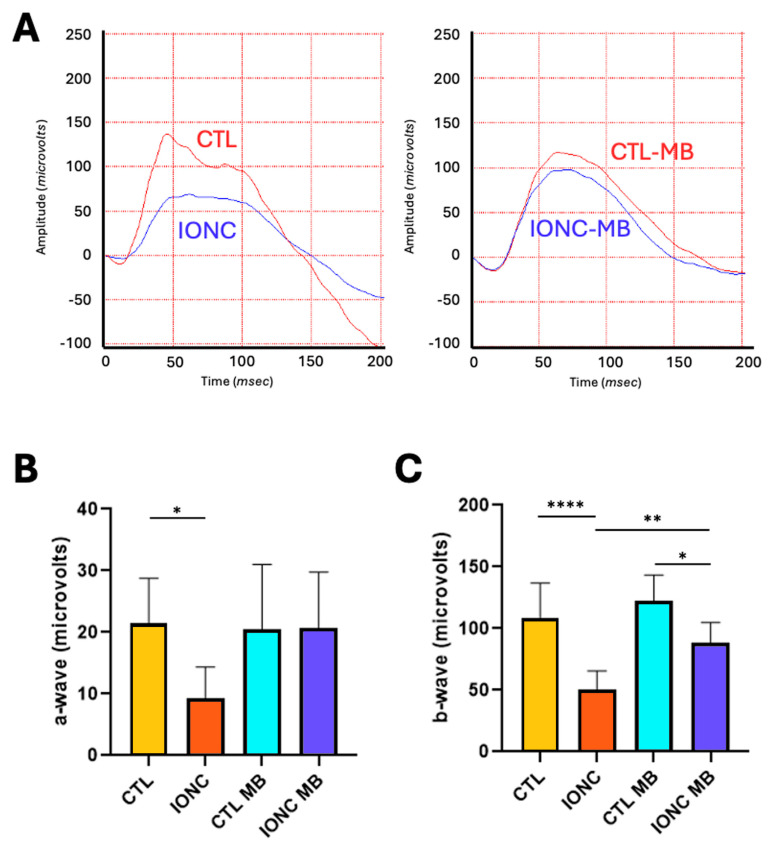
MB prevents IONC-induced changes in the scotopic ERG. (**A**) Representative electroretinograms taken 21 days post-surgery for animals that received either PBS or MB. The red line corresponds to the right eye, whereas the blue line is the recording of the left eye. (**B**) Amplitude of the a-wave in the four experimental groups. (**C**) Amplitude of the b-wave in the four experimental groups. In both cases, IONC induced a significant decrease in the a- and b-wave compared to control (CTL), whereas MB partially or completely prevented it. Each bar represents the mean ± SEM of eight animals. One-way ANOVA followed by Bonferroni post hoc test. Asterisks indicate significant differences: * *p* < 0.05; ** *p* < 0.01; **** *p* < 0.0001.

Scotopic ERG showed that those eyes subjected to the IONC procedure had a significant reduction of both the a-wave (*p* = 0.0356) and the b-wave (*p* < 0.0001) compared to CTL eyes. Treatment with MB of sham-operated eyes (CTL-MB) had no impact on these parameters. In the case of the a-wave, a significant recovery by MB treatment could not be demonstrated, although the IONC-MB group was undistinguishable from the CTL-MB group (Figure 2A,B). On the other hand, MB treatment (IONC-MB) resulted in a significant recovery of the b-wave compared to IONC (*p* = 0.0055), although it did not reach the complete amplitude shown by CTL-MB (*p* = 0.0180) (Figure 2A,C).

#### 2.2.2. Scotopic Oscillatory Potentials

**Figure 3:**
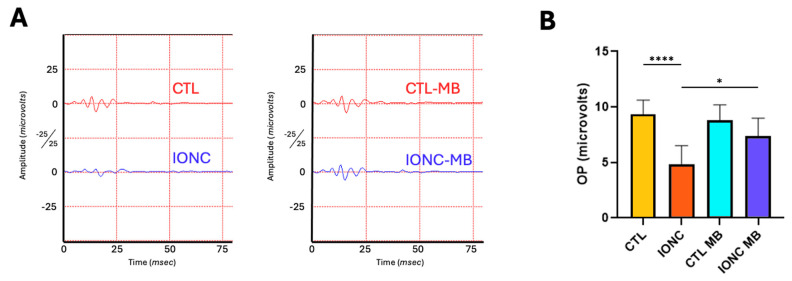
MB prevents IONC-induced changes in the oscillatory potentials. (**A**) Representative scotopic oscillatory potentials (OP) of the ERG taken 21 days post-surgery for animals that received either PBS or MB. The red line corresponds to the right eye whereas the blue line is the recording of the left eye. (**B**) Sum of amplitudes of the OP in the four experimental groups. IONC induced a significant decrease in the OP compared to control (CTL), whereas MB treatment significantly prevented it. Each bar represents the mean ± SEM of eight animals. One-way ANOVA followed by Bonferroni post hoc test. Asterisks indicate significant differences: * *p* < 0.05; **** *p* < 0.0001.

Oscillatory potentials (OP) of eyes subjected to IONC showed a significant (*p* < 0.0001) reduction of amplitude and complexity of the OP patterns compared to controls (CTL) (Figure 3A,B). MB treatment in IONC-operated eyes (IONC-MB) managed to produce a significant (*p* = 0.0102) preservation of these parameters compared to the IONC group, to the point of becoming undistinguishable from the CTL-MB control (Figure 3A,B). As with other parameters, MB treatment had no effect on the OPs of sham-operated eyes.

#### 2.2.3. Photopic Full Field Electroretinography

**Figure 4:**
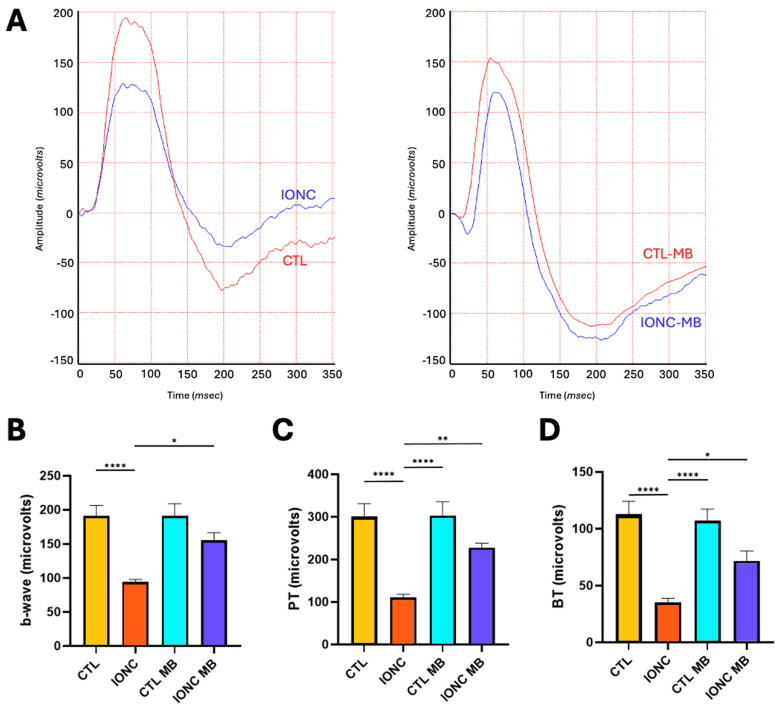
MB prevents IONC-induced changes in the photopic ERG. (**A**) Representative electroretinograms taken 21 days post-surgery for animals that received either PBS or MB. The red line corresponds to the right eye, whereas the blue line is the recording of the left eye. (**B**) Amplitude of the b-wave in the four experimental groups. (**C**) Amplitude of the PhNR wave measured as PT in the four experimental groups. (**D**) Amplitude of the PhNR wave measured as BT in the four experimental groups. In all three cases, IONC induced a significant decrease in the b-wave and PhNR compared to control (CTL), whereas MB significantly prevented it. Each bar represents the mean ± SEM of eight animals. One-way ANOVA followed by Bonferroni post hoc test. Asterisks indicate significant differences: * *p* < 0.05; ** *p* < 0.01; **** *p* < 0.0001.

To obtain a more objective measurement of RGC function, the amplitude of the photopic b-wave as well as the photopic negative response (PhNR) were evaluated through a full field photopic ERG. The behavior of the photopic b-wave was almost identical to that described for the scERG. IONC surgery resulted in a very significant (*p* < 0.0001) reduction of amplitude in comparison to CTL, whereas MB treatment (IONC-MB) resulted in a significant (*p* = 0.0145), and complete, preservation of amplitude when compared to IONC (Figure 4A,B).

As for the PhNR, peak-to-trough (PT, Figure 4C) and baseline-to-trough (BT, Figure 4D) measurements were obtained. Both methods showed a marked (*p* < 0.0001) reduction of amplitude in the IONC group when compared to controls (CTL). Treatment with MB (IONC-MB) significantly preserved the amplitude for both PT (*p* = 0.0068) and BT (*p* = 0.0442) methods. No statistically significant differences were found between the IONC-MB and the CTL-MB groups.

#### 2.2.4. Pattern Electroretinography

**Figure 5:**
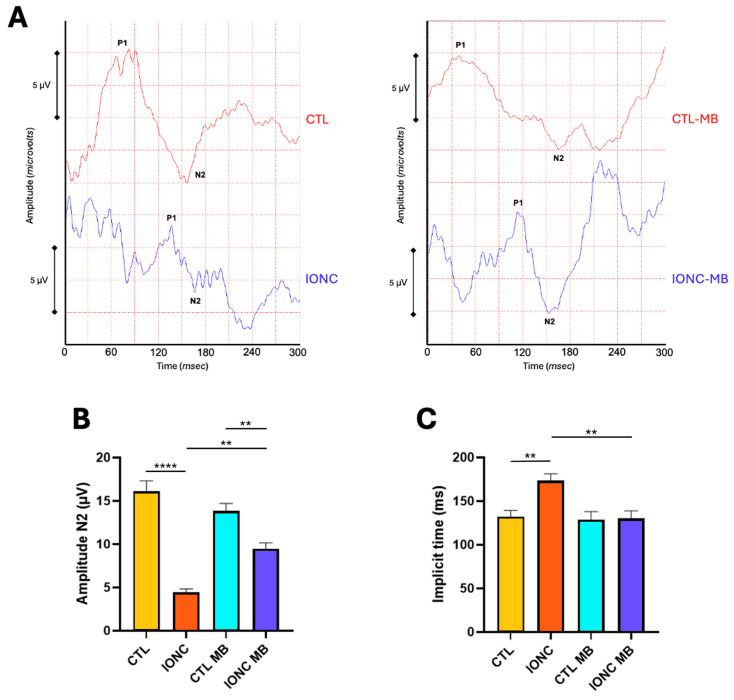
MB prevents IONC-induced changes in the PERG. (**A**) Representative pattern electroretinograms taken 21 days post-surgery for animals that received either PBS or MB. The red line corresponds to the right eye, whereas the blue line is the recording of the left eye. (**B**) Amplitude of the N2 wave in the four experimental groups. (**C**) N2 implicit time in the four experimental groups. IONC induced a significant decrease in the N2 amplitude compared to the control (CTL), whereas it increased the N2 implicit time. MB treatment partially restored N2 amplitude while completely restored its implicit time. Each bar represents the mean ± SEM of eight animals. One-way ANOVA followed by Bonferroni post hoc test. Asterisks indicate significant differences: ** *p* < 0.01; **** *p* < 0.0001.

PERG was also recorded to evaluate with higher sensitivity RGC function and integrity. Two main parameters were evaluated because of their relevance in neuropathy assessment: amplitude and implicit time of the N2 wave. Eyes subjected to IONC surgery had a very significant (*p* < 0.0001) reduction of N2 amplitude compared to CTL (Figure 5A,B). MB treatment (IONC-MB) resulted in a significant preservation of amplitude compared to IONC (*p* = 0.0014), although it did not completely recover when compared to CTL-MB (*p* = 0.0060) (Figure 5A,B).

Regarding N2 implicit time, it was significantly prolonged in IONC operated eyes in comparison to CTL (*p* = 0.0087). This latency was significantly (*p* = 0.0057) reduced in the IONC-MB group when compared to non-treated eyes (IONC) (Figure 5A,C).

### 2.3. Methylene Blue Prevents IONC-Induced Cell Death in the Ganglion Cell Layer

**Figure 6:**
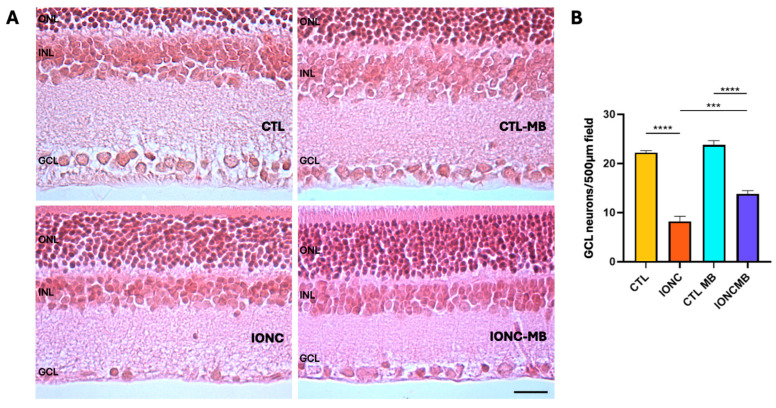
MB prevents IONC-induced cell death in the GCL. Representative histological images of the retina of animals of the four experimental groups, taken 21 days after surgery, and stained with hematoxylin and eosin (**A**). Three layers of the retina are labeled in the pictures for reference: outer nuclear layer (ONL), inner nuclear layer (INL), and GCL. Scale bar = 50 μm. Quantification of the number of neurons in the GCL (**B**). Bars represent the mean ± SEM of all samples (6 eyes, 7 fields per eye, for a total of 42 fields per group). One-way ANOVA followed by Bonferroni post hoc test. Asterisks represent statistically significant differences. *** *p* < 0.001; **** *p* < 0.0001.

Histological sections of the retina were analyzed (Figure 6A) and the number of viable nuclei in the ganglion cell layer (GCL) were recorded (Figure 6B). Eyes subjected to IONC showed a very significant (*p* < 0.0001) loss of GCL neurons in comparison to controls (CTL). Treatment with MB of sham-operated eyes (CTL-MB) had no impact on this parameter. However, MB treatment applied to IONC operated eyes (IONC-MB) resulted in a significant (*p* = 0.0003) preservation of neurons in relation to IONC. Nevertheless, the treatment could not completely prevent cell death in the GCL, whose number was still significantly lower (*p* < 0.0001) when compared to the proper control (CTL-MB).

## 3. Discussion

Trauma to the optic nerve can be direct when a penetrating injury damages the axons in an almost irreversible manner or, more frequently, indirectly through an injury to the head or the periocular tissues whose mechanical forces apply traction on some point of the visual pathway, damaging the axons. The latter is fortunately the most common occurrence and has a better probability of spontaneous recovery [2]. However, there can be chronic axonal degeneration even after a single blunt trauma [34]. Secondary mechanisms of degeneration, such as neuroinflammation [35,36] and oxidative stress [37,38], or even local complications, such as optic nerve edema, hematoma or arterial occlusion/compression resulting in optic nerve head ischemia, can perpetuate axonal degeneration distant to the injury via anterograde [39] or retrograde [40,41] mechanisms. Recent studies have shown evidence of a critical period for remodeling and recovery encompassing 14 to 30 days after injury [34]. Furthermore, when some degree of spontaneous recovery occurs in the clinical setting, it mostly happens during the first month after injury [42]. For that reason, developing neuroprotective strategies that could potentially improve those repairing mechanisms during that critical window becomes crucial in preventing trauma-related blindness.

The current treatment strategy for TON consists of either observation without intervention or treatment with corticosteroids [43]. In a few cases, when compartment syndrome of the anterior optic nerve occurs secondary to subdural or subarachnoid hematoma, surgery can be an option [2]. However, there is no hard evidence demonstrating that any treatment is better than observation alone [1,13]. Although several experimental strategies, such as treatment with erythropoietin [44,45,46,47,48], intravitreal BDNF and cAMP [49], up-regulation of mTOR [14], trans-corneal electrical stimulation [50,51,52], hypothermia [23] and hypothermia mimetic molecules [24], are under study, none of them has yet reached the clinic.

One important aspect that separates the use of MB from other neuroprotective treatments under study is that MB is a drug already approved for clinical use for conditions such as methemoglobinemia [53] and norepinephrine refractory hypotension [54], among others. It has a high safety profile [55] and is an extremely affordable and accessible drug given that it is part of the World Health Organization’s List of Essential Medicines. So, finding a new indication, such as neuroprotection, for an already approved drug with these characteristics could have a major impact in the management of several neurodegenerative conditions worldwide [56].

In this study, we have demonstrated that MB treatment exhibits neuroprotective properties that allow the prevention and mitigation of the impact of TON progression on neuronal integrity and function. This study follows our previous work, where we have shown that MB had a beneficial impact in the setting of perinatal asphyxia-induced retinopathy [28,29] and, most recently, in glaucoma [30], which is the most common cause of optic neuropathy. Both pathologies share with TON many pathophysiological mechanisms of neuropathy progression, such as oxidative stress [37,38,57] and axonal neurodegeneration [58]. These findings may take us one step further into developing effective neuroprotective treatments to prevent the progression of retinopathies and neuropathies in general, and therefore, to reduce the burden of irreversible blindness. In order to do so, the pathophysiological effects of the IONC procedure were thoroughly measured, as well as the therapeutic effect of MB on retinal and optic nerve function. Several types of electroretinography were used, and a very detailed picture of the integrity and function of retinal circuits was acquired, while progressively focusing into RGCs and the optic nerve.

Full-field scERG showed that IONC produced a significant reduction in the amplitude of both the a- and b-waves. These waves represent the electrical currents originated in photoreceptors’ and in the bipolar/Müller glia cells’ activity, respectively [59]. Given that the IONC model produces direct compression to the axons of the RGCs, it is most likely that these findings are due to two reasons: (i) the traumatic insult to the RGCs and their eventual demise produces electrical disruptions of the retinal circuitry that impact on the preceding neurons; (ii) compression to the optic nerve is accompanied by temporary occlusion of the central retinal artery, and therefore, ischemia to the inner retina occurs (as it happens in human TON due to optic nerve edema, inflammation or hematoma, among other complications [2]). This phenomenon has already been described by our group as a common feature of inner retinal insult and optic neuropathy [23,24,28,29,30,60,61]. The treatment with MB resulted in a significant, but partial, preservation of the b-wave, while the a-wave did not show a significant recovery. This suggests that MB produced a significant restoration of activity of the electrical circuits generated by bipolar/Müller glia cells, probably through reducing neuronal stress. Obviously, a clear gradient of damage towards the inner retina was still evident after the treatment, as has been shown in other models [30].

Scotopic OP served as a useful parameter to evaluate even further into the physiology of the inner retina. OPs are originated from bipolar and amacrine neurons [62,63] and are known to be a very sensitive parameter of inner retinal vascular dysfunction and ischemia/hypoxia [62,64,65]. In line with previously stated effects of the IONC model on retinal perfusion, OPs were significantly altered in IONC-operated eyes, while MB treatment managed to fully restore their amplitude and waveform complexity. This finding would suggest a protective effect over neurons suffering from an ischemic microenvironment. These results are also in line with our recent description on the effects of MB over the OPs in glaucomatous neuropathy [30].

To evaluate the function of RGCs more objectively and with higher sensitivity, two other ERG modalities were performed: phERG, with special attention to the PhNR, and PERG. The PhNR is a negative deflection of the full-field phERG that comes after the b-wave and is fully originated by the RGCs. Evaluation of the PhNR constitutes a very valuable tool in the study of the innermost retina and many optic neuropathies [66,67]. In our experiment, the PhNR was significantly reduced in IONC eyes in comparison to controls, meaning that a clear affection of the RGCs was generated by this traumatism. However, the administration of MB clearly prevented this reduction to the point that no significant differences were found with the CTL-MB controls. The photopic b-wave showed results comparable to those of the scERG previously discussed. Afterwards, PERG was performed to improve RGC assessment and to quantify the N2-wave’s amplitude and implicit time. The latter parameter is known to be even more sensitive than the N2’s amplitude and to have some correlation with neuronal stress and delayed electrical conduction [68]. The amplitude of the N2 wave (equivalent to human N95 and originated from RGCs [69]) was significantly diminished by the IONC surgery compared to controls and, as expected, its implicit time was significantly prolonged. Both parameters were significantly preserved by MB treatment in the IONC-MB group, although the recovery of the N2 wave amplitude was not complete, suggesting that some neuronal stress was still present. All these data would indicate a clear neuroprotective effect elicited by MB, by preserving RGC integrity and function, and consequently restoring healthier electrical currents on the retinal circuitry.

Histological evaluation of retinal sections further corroborated the electrophysiological data by showing a very significant loss of neurons in the GCL of the IONC group in comparison to controls. These morphological hallmarks coincided with the electrophysiological findings, for all ERG modalities. Treatment with MB translated into a highly significant restoration of this parameter supporting the relevance of adding this drug to the current treatments for TON neuroprotection.

The World Health Organization’s List of Essential Medicines includes MB as one of the most effective and safe medicines required in a healthcare system [70]. It has been approved for use in the clinical treatment of various pathologies [53,54,71]. Considering our findings in the IONC model, as well as those from other models of ocular pathologies [28,30], it would be interesting to evaluate its potential clinical use in treating degenerative ocular pathologies. The use of MB could also benefit other pathologies involving the NOS/GC/cGMP cascades and cellular damage induced by reactive nitrogen species [72].

This study has some limitations. First, this work was designed as a proof-of-concept, so experiments were carried out exclusively in male rats to reduce potential variability due to sex and hormonal influences. Future studies will show whether these hormones have any influence in the efficacy of the MB treatment. On the other hand, this experiment was a first approach in determining the potential effects of MB in the progression of TON, so a short treatment was planned. However, as previously stated, the window of opportunity for optimizing neuronal protection and remodeling may occur during the first month after injury [34,42], so future studies implementing a prolonged MB treatment might offer even better results.

## 4. Materials and Methods

### 4.1. Traumatic Neuropathy Model Through Intraorbital Optic Nerve Crush (IONC)

Male, young (8-week-old) Wistar rats (n = 32) with genetic quality and sanitary certification from the animal facility of our Institution were cared for in accordance with guidelines published in the ARVO Statement for the Use of Animals in Ophthalmic and Vision Research. All procedures were approved by the Ethical Committee of CICUAL (Comité Institucional para el Uso y Cuidado de Animales de Laboratorio, Facultad de Medicina, Universidad de Buenos Aires, Resolution RESCD-2023-2359-E-UBA-DCT#FMED). Animals were kept under standard laboratory conditions, with light/dark cycles of 12/12 h, constant temperature of 21.0 ± 2.0 °C, and food and water provided ad libitum.

The IONC protocol was performed as described [73], with slight modifications. Briefly, a small incision was performed in the superior temporal quadrant of the left eye, and eyeball proptosis was performed to provide access to the optic nerve. The optic nerve was crushed with forceps for 60 s at 1.5 mm from the ocular globe, and the incision was closed with suture. The contralateral eye was sham-operated: the optic nerve was exposed but not crushed. After surgery, intraperitoneal Tramadol 5 mg/kg (John Martin S.R.L., Buenos Aires, Argentina) and topic ophthalmic erythromycin ointment (Eritromicina Elea, Elea, Buenos Aires, Argentina) were applied as analgesic and antibiotic prophylaxis, respectively. Animals were checked daily for signs of pain or discomfort.

Immediately after surgery, animals were randomly distributed into two groups. Half of the rats (n = 16) started intraperitoneal treatment with 2.0 mg/kg MB (Sigma, St. Louis, MO, USA), immediately post-surgery and at 6, 12, 18 and 24 h after surgery. The MB dose of 2 mg/kg was selected in agreement with the range of doses used in the clinical practice in humans (1–2 mg/kg) [74,75], and it has already been administered to rats for the treatment of ocular conditions [28,29,30]. This dose is known to be safe, effective and deprived of toxicity [76]. The frequency of administration of the MB was determined according to the half-life of the drug [77]. Then, animals were left untreated for three weeks. At day twenty-one after surgery, scotopic oscillatory potentials (OP), scotopic full field (scERG), photopic full field (phERG) and pattern (PERG) electroretinography were performed for retinal function assessment (see below). After PERG evaluation, all animals were sacrificed (Figure 1). Eyes were enucleated and retinal tissue were dissected, fixed and paraffin embedded for morphological analysis (see below).

### 4.2. Scotopic Full-Field Electroretinography (scERG) and Scotopic Oscillatory Potentials (OP)

Twenty-one days after surgery, the first group of 8 rats (n = 16 eyes, 8 eyes per group) was subjected to scotopic electroretinography, as described [78]. Briefly, after an overnight adaptation in the dark, rats were anesthetized with a solution of ketamine (40 mg/kg) (Ketamina 50, Holliday-Scott S.A., Buenos Aires, Argentina) and xylazine (5 mg/kg) (Xilacina 20 Richmond, Laboratorios Richmond, Buenos Aires, Argentina) under dim red illumination. An ophthalmic solution containing 5% phenylephrine hydrochloride and 0.5% tropicamide (Fotorretin, Poen, Buenos Aires, Argentina) was used to dilate the pupils, and a local proparacaine ointment (Poen-caina, Poen) was applied over the cornea as a local anesthetic. Rats were placed facing the stimulus at 25 cm in a highly reflective environment. Using a commercially available ERG equipment and software (Akonic S.A., Buenos Aires, Argentina, http://www.akonicsa.com/ (accessed on 16 June 2025)), scotopic electroretinograms (scERG) were recorded from both eyes simultaneously, and 10 responses were collected to flashes of unattenuated white light (1 ms, 1 Hz) from a photic stimulator set at maximum brightness. The registered response was amplified (9 cd s/m^2^ without filter), filtered (1.5 Hz low-pass filter, 300 Hz high-pass filter, notch activated), and averaged. Total time of analysis was 500 ms. The a-wave was estimated as the difference in amplitude between the recording at onset and the trough of the negative deflection, and the b-wave amplitude was calculated from the trough of the a-wave to the following peak. To calculate oscillatory potentials (OP), the same photic stimulator was used with filters of high (1000 Hz) and low (100 Hz) frequency. Flashes of white light were delivered at maximum brightness (1 ms, 0.1 Hz), and 5 responses were averaged. The amplitudes of the OPs were recorded by using the peak-to-trough method.

### 4.3. Photopic Full Field Electroretinography (phERG)

Twenty-one days after surgery, another group of 8 rats (n = 16 eyes, 8 eyes per group) was subjected to photopic electroretinography, as described [79], with slight modifications. Briefly, rats were adapted to the environmental light (background white light; ~90 lux) for a minimum of 30 min and then anesthetized as above. Pupil dilatation and local anesthesia were also performed as above. Rats were placed facing the stimulus at 25 cm in a highly reflective environment. Using the same ERG equipment and software, photopic electroretinograms (phERG) were recorded from both eyes simultaneously, and 10 responses were collected to flashes of unattenuated white light (1 ms, 1 Hz) from a photic stimulator set at maximum brightness after a pre-conditioning of 8 non-averaged flashes. The registered response was amplified (9 cd s/m^2^ without filter), filtered (1.5 Hz low-pass filter, 500 Hz high-pass filter, notch activated), and averaged. Total time of analysis was 500 ms. The b-wave amplitude was calculated from the trough of the a-wave to the following peak. The photopic negative response, which is the negative deflection following the b-wave, was measured using two paradigms as described [66]. BT (baseline to trough) was measured from ERG baseline to the minimum point in the trough after the b-wave, while PT (peak-to-trough) was measured from the peak of the b-wave to the previously mentioned trough.

### 4.4. Pattern Electroretinography (PERG)

Immediately after phERG evaluation the same group of 8 rats (n = 16 eyes, 8 eyes per group) were subjected to pattern electroretinography, as described [80], with slight modifications. An additional injection of a fraction of the initial dose of anesthetic solution was given, if necessary, to maintain animals anesthetized during the whole electroretinographic assessment. The anesthesia used does not affect amplitude of the ERG responses [81,82]. An extra drop of local proparacaine ointment was applied over the cornea. Rats were placed at a 45° angle, so one of their eyes was facing the stimulus at 20 cm. Transient PERG were recorded from each eye separately. The visual stimulus was generated by commercial software (Akonic S.A.) and displayed in a CRT monitor (SyncMaster 591s, Samsung, Buenos Aires, Argentina). It consisted of a black and white 10 × 8 checkerboard with a 50% duty cycle that alternated at a temporal frequency of 2 reversals per second (1.00 Hz) and a spatial frequency of 0.068 cycles per degree. Contrast was maintained at 90%, and mean luminescence of the projected display was 50 cd/m^2^. Duration of the stimulus was 300 milliseconds, responses were filtered (1.5 Hz low-pass filter, 500 Hz high-pass filter, notch activated) and 100 cycles were averaged. N2 wave amplitude was calculated as the difference between the P1 peak and the following trough. N2 peak latency is the time from the initiation of the stimulus until the N2 trough occurs.

### 4.5. Histology and Morphological Evaluation

After completing electroretinography, rats (n = 6 per group) were anaesthetized with a lethal dose of ketamine/xylazine and sacrificed by decapitation. Eyes were enucleated and carefully dissected under the microscope to remove all extraocular tissues, cornea and lens leaving the complete posterior eye segment. Optic cups were fixed in 4.0% paraformaldehyde for 48 h at 4 °C, dehydrated, paraffin embedded, and sectioned (3.0 μm thick). Sections were stained with hematoxylin and eosin. Before assays, care was taken in selecting anatomically matching areas among animals for an accurate analysis. To avoid variations in the quantification process, all photographs were obtained the same day and under the same light and contrast conditions and by the same operator (RN). Photographs were obtained with a Zeiss Axiolab RE upright trinocular microscope (Carl Zeiss, Oberkochen, Germany) coupled with an Olympus Q-Color 3 camera and acquired with Q-Capture Pro software version 6.0 (Olympus America Inc., Center Valley, PA, USA). Images of retinal sections were obtained under the 10× objective lens with an acquirement resolution of 2080 × 1542 (final resolution was equivalent to a 200× magnification power). Manual counting of neuronal nuclei in the GCL was performed for each experimental group in 7 fields per eye (6 eyes per group, for a total of 42 fields per group), as previously reported [25]. Neuronal nuclei were identified based on morphological parameters: large pale nucleus with prominent nucleolus. A nucleus diameter of 15–20 μm was considered for counting based on size differences reported between neurons and glial cells [83], as well as between RGC and displaced amacrine neurons [84,85,86,87]. Glial cells are recognized for their smaller size with darker stained nucleus. Quantification of these parameters was performed manually using ImageJ software, version 1.53g (Wayne Rasband & Co., National Institutes of Health, Bethesda, MD, USA) in all eyes.

### 4.6. Statistical Analysis

Data were analyzed with GraphPad Prism software, version 10.2.3, and were considered statistically significant when *p* < 0.05. Values are expressed as means ± SEM. All data sets were evaluated for normality (Shapiro–Wilk) and homoscedasticity (Levene). Since all datasets followed a normal distribution, they were evaluated by one-way ANOVA followed by Dunnet’s (Bonferroni) post hoc test.

## 5. Conclusions

In this work, we have shown evidence of MB having neuroprotective properties in an experimental model of TON. MB was able to successfully preserve the number of GCL neurons, as well as several electrophysiological parameters of retinal function. In the future, this drug could potentially serve as an effective treatment for preserving visual function in patients with trauma-related visual complications.

## Data Availability

The original contributions presented in this study are included in the article. Further inquiries can be directed to the corresponding authors.

## Abbreviations

The following abbreviations are used in this manuscript:

ANOVA	Analysis of variance
BDNF	Brain-derived neurotrophic factor
BT	Baseline-to-trough
cAMP	Cyclic adenosine monophosphate
CTL	Control
ERG	Electroretinogram
INL	Inner nuclear layer
IONC	Intraorbital nerve crush
GCL	Ganglion cell layer
MB	Methylene blue
NO	Nitric oxide
NOS	Nitric oxide synthase
ONL	Outer nuclear layer
OP	Oscillatory potentials
PBS	Phosphate buffered saline
PERG	Pattern electroretinography
phERG	Photopic electroretinography
PhNR	Photopic negative response
PT	Peak-to-trough
RGC	Retinal ganglion cells
scERG	Scotopic electroretinogram
TON	Traumatic optic neuropathy
